# Investigation of the Physical Properties of Poly(ester–ether)s and Multi-Walled Carbon Nanotube Nanocomposites

**DOI:** 10.3390/ma19163397

**Published:** 2026-08-10

**Authors:** Giulia Guidotti, Franco Dominici, Daria Armani, Marco Rallini, Mauro Zanuccoli, Claudio Fiegna, Debora Puglia, Nadia Lotti

**Affiliations:** 1 Department of Civil, Chemical, Environmental and Materials Engineering, University of Bologna, Via Terracini 28, 40131 Bologna, Italy; giulia.guidotti9@unibo.it (G.G.); daria.armani2@unibo.it (D.A.); nadia.lotti@unibo.it (N.L.); 2 Department of Civil and Environmental Engineering, University of Perugia, Strada di Pentima 4, 05100 Terni, Italy; franco.dominici@unipg.it (F.D.); marco.rallini@unipg.it (M.R.); 3 Interdepartmental Centre for Industrial Agri-Food Research, CIRI-AGRO, University of Bologna, Via Quinto Bucci 336, 47521 Cesena, Italy; 4 Department of Electrical Energy and Information Engineering, University of Bologna, Via Dell’ Università 50, 47522 Cesena, Italy; mauro.zanuccoli@unibo.it (M.Z.);; 5 Interdepartmental Centre for Industrial Research on Advanced Applications in Mechanical Engineering and Materials Technology, CIRI-MAM, University of Bologna, Viale del Risorgimento 2, 40136 Bologna, Italy

**Keywords:** carbon nanotubes, masterbatch, polybutylene terephthalate, polyester–polyether block copolymer, strain sensor

## Abstract

This work describes the design and characterization of nanocomposites based on multi-walled carbon nanotubes (MWCNTs) and commercial polymer matrices for innovative electronic applications. This work addresses the need for advanced materials for flexible electronics, sensing, and electromagnetic shielding. Sipolprene^®^ 25170-W, a flexible and durable polyester–polyether block copolymer, was used as the matrix. For filler incorporation, the commercial masterbatch Plasticyl™ PBT-1501 (15 wt% of MWCNTs in PBT, polybutylene terephthalate) was employed, ensuring operational safety and ease of dispersion. The samples were produced as films (with masterbatch contents ranging from 10% to 30% corresponding to a MWCNT content ranging from 1.5 wt% to 4.5 wt%) via twin-screw extrusion with a flat die. Characterization included SEM, FT-IR, TGA, DSC, tensile testing, surface wettability, volume resistivity measurements, and electro-mechanical tests. All the results confirmed good dispersion of the filler within the matrix: from a mechanical point of view, the addition of MWCNTs increased the Young’s modulus from 25 MPa of the neat material to 122 MPa of the material containing 4.5 wt% of MWCNTs, enhancing stiffness while maintaining good film handleability. Thermal analysis revealed the high stability of the obtained system and allowed us to identify the appropriate processing temperature parameters to guarantee the thermal stability of the materials during processing. Finally, electrical tests demonstrated a significant reduction in volume resistivity with increasing filler content: the volume resistivity decreased by about eleven orders of magnitude, from approximately 10^8^ Ohm × cm of the unmodified material to 10^−3^ Ohm × cm for the material containing 4.5 wt% of MWCNTs. the sample with 30% of filler exhibited the typical behavior of a conductive material, and it was demonstrated that it could be used as an in situ strain sensor. All these findings confirm the potential of the developed materials for advanced technological applications.

## 1. Introduction

Discovered in 1991, carbon nanotubes (CNTs) are an allotrope of carbon consisting of graphene sheets rolled into cylindrical nanostructures with nanometric diameters and lengths up to the millimeter scale, making them nearly one-dimensional systems [1]. They are mainly classified into single-walled carbon nanotubes (SWCNTs) and multi-walled carbon nanotubes (MWCNTs) [2]. Due to their exceptional mechanical, electrical, and thermal properties, they are widely used in various fields, including sensors, energy systems, electronics, environmental protection, and electromagnetic shielding, and are also highly promising materials for advanced applications, including nanoelectronics [3]. In more detail, from a mechanical point of view, they exhibit extremely high mechanical strength—it has been estimated that the Young’s modulus of a nanotube can reach 4 TPa, while its tensile strength can reach about 220 GPa, a value about 100 times higher than steel, together with variable electrical behavior (metallic or semiconducting), which can be further tuned through doping. However, CNTs present challenges related to dispersion, due to their tendency to aggregate and their hydrophobic nature. To overcome this application limit, the most common solution is to disperse and distribute the nanotubes through both physical and chemical methods, according to the requirements of the final device that needs to be manufactured [4]. Physical approaches, such as high-power ultrasonication, temporarily break nanotube agglomerates. The conjugated structure of the nanotube is not affected, and therefore all the structural properties of the nanotube itself can be maintained. However, re-aggregation occurs once the energy input is removed. Chemical methods, both non-covalent and covalent, provide more stable dispersion and enable the use of nanotubes as reactive components in high-performance nanocomposites [5].

The design and creation of polymer nanocomposites based on carbon nanotubes have led to the development of a series of materials with advanced properties compared to common polymers [6]. This strategy allows them to overcome the limit of inefficient dispersion of the nanotubes, treating the polymer as if it was a normal solid dispersing medium. At the same time, the use of MWCNTs inside polymer-based nanocomposites would also solve the issues related to their potential adverse effects on human health and the environment [7], making it possible to obtain new materials with improved synergistic properties and safety [8,9]. In particular, the use of polyester-based matrices offers a dual advantage: on the one hand, it enables intrinsically insulating materials to become electrically conductive; on the other hand, when the starting matrix is flexible, it can mitigate the inherent rigidity of carbon nanotubes, resulting in materials that are easier to process and handle, and potentially suitable for a wide range of applications. Several studies report simple and effective strategies to produce conductive polyester-based nanocomposites by incorporating carbon fillers such as CNTs, exfoliated graphite, or carbon black. If aromatic polyesters are considered, several results on the electrical behavior of terephthalic acid-based materials have been widely discussed in the literature as a function of temperature, frequency, and crystallinity degree [10,11,12,13]. In poly(ethylene terephthalate)- and poly(butylene terephthalate)-based composites, for example, these fillers improved thermal stability and electrical conductivity [14,15]. Moreover, experimental tests have demonstrated the possibility of producing composite nanofibers of poly(butylene terephthalate), PBT, and MWCNTs through an electrospinning technique in the form of a network of random fibers [16].

A study at the Technical University of Liberec in the Czech Republic about the electrical properties of composites formed by a PBT matrix and carbon nanotubes was published in 2018 [17,18]. The filler used consisted of multi-walled carbon nanotubes stabilized in the form of a commercial masterbatch called Plasticyl™ PBT-1501.

**Figure 1:**
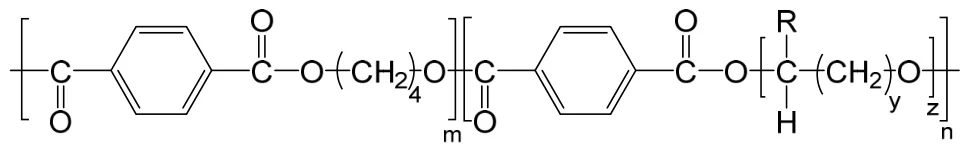
Chemical structure of Sipolprene^®^.

The use of CNT masterbatches represents an effective alternative to conventional dispersion techniques, as it facilitates a more homogeneous distribution of the nanotubes while simplifying processing and reducing the handling of pristine nanomaterials. As an example, Zhu et al. [19] developed a thermoplastic electrically conductive polyurethane (TPU)/single-walled carbon nanotube nanocomposite by incorporating a SWCNT masterbatch based on a polyol ester maintaining the low viscosity and good processability of the nanocomposites. Arnal et al. [20] used a polyamide 6 (PA6)-based MWCNT masterbatch to make an electrically conductive polyamide 12 (PA12) with analogous results. Following a similar approach, in this study we explore the use of Plasticyl™ PBT-1501 for the modification of a flexible copolymer that is chemically compatible with the masterbatch in order to achieve effective nanotube dispersion through a scalable and industrially relevant processing route. In more detail, the polymeric class investigated in this study consisted of commercial Sipolprene^®^ copolymers. These poly(ether–ester)s (PEEs) are thermoplastic copolymers composed of rigid and highly crystalline blocks of PBT and amorphous elastomeric blocks made of poly(ester–ether) units of different natures (Figure 1).

As a source of carbon nanotubes, we chose a commercial formulation, Plasticyl™-PBT-1501, consisting of MWCNTs dispersed at 15% by weight in a PBT matrix. These two components were selected because the former, due to its peculiar molecular architecture and the presence of polyether segments, exhibits high chemical resistance, excellent mechanical flexibility, and good processability. Moreover, since PBT was also present in the masterbatch used as a filler, a high level of compatibility between the filler and the matrix was expected, thereby ensuring the optimal functional properties of the resulting nanocomposites. The analysis of the electrical behavior and mechanical performance of the composites as a function of the weight of MWCNTs constituted one of the starting points of the present work, which focuses on the design, preparation, and subsequent characterization of nanocomposites based on multi-walled carbon nanotubes and commercial polymer matrices, with the aim of developing innovative materials with enhanced electronic properties.

This aspect is particularly important when the material is intended for flexible electronic applications, such as piezoresistive sensors for strain monitoring, or electromagnetic interference (EMI) shielding. In fact, good dispersion and distribution of conductive particles embedded in the matrix promote the formation of effective conductive pathways, thereby reducing the filler content required to achieve the electrical percolation threshold [21]. This issue becomes even more critical in polymer blends, in which fillers tend to be predominantly distributed within a specific phase or the distribution is concentrated at the interface between the two polymers [22]. In this context, the combination of Sipolprene^®^ copolymers and Plasticyl™ PBT-1501 represents a promising solution owing to the high compatibility between the polymer matrix and the conductive masterbatch, which facilitates a more uniform filler distribution and the development of stable conductive pathways.

To the best of the authors’ knowledge, no previous studies have reported the use of Sipolprene^®^ copolymers combined with carbon nanotube-based conductive masterbatches for the development of flexible conductive composites. As a consequence, modified Sipolprene^®^ may be a feasible choice as the replacement of widely studied traditional elastomeric conductive materials, such as silicone [23,24], polyurethane [25,26], or ethylene propylene diene M-class rubber (EPDM) [27], which have been extensively investigated for electronic and sensing applications. The nanocomposites were obtained through cast extrusion and fully characterized for their thermal, mechanical, surface, and electrical properties, comparing the experimental results with those obtained from the virgin polymer material in relation to the amount of filler used [28]. This study introduces an innovative approach to developing flexible, electrically conductive polymer nanocomposites for advanced electronic and sensing applications. Instead of handling pristine nanomaterials (which often pose dispersion and aggregation challenges), this research leverages a PBT-based masterbatch that ensures high chemical compatibility with the PBT segments in the Sipolprene matrix. By utilizing a flexible polyester-based matrix, this approach successfully mitigates the inherent mechanical rigidity of carbon nanotubes while imparting electrical conductivity to an intrinsically insulating polymer. Modified Sipolprene emerges as a viable, highly processable, and industrially scalable alternative to traditional elastomeric conductive materials.

## 2. Materials and Methods

### 2.1. Materials

The polymeric matrix of Sipolprene 25170-W was kindly provided by Sipol Spa (Mortara, Pavia, Itay) in the form of pellets. From the code and the technical data sheet provided by the producers, it was possible to identify a priori the maximum melting temperature corresponding to 173 °C, while the letter W represented the presence of additives, such as stabilizers for UV radiation. The 15 wt% MWCNT (NC7000™) masterbatch in the PBT matrix called Plasticyl™ PBT-1501 was kindly donated by Nanocyl SA (Sambreville, Belgium). NC7000™ has the following characteristics: diameter of 9.5 nm, aspect ratio in the range of 100–1000:1, specific surface area in the range of 250–300 m^2^/g, volume resistivity of 10^−4^ Ω cm, thermal conductivity > 3000 W/mK, and tensile strength in the range of approximately 10–60 GPa. The main technical data regarding these two components are summarized in Table 1.

### 2.2. Methods

The nanocomposite films were prepared starting from a Sipolprene matrix and varying amounts of Plasticyl, ranging from 10 wt% to 30 wt% (corresponding to a final CNT amount ranging from 1.5 wt% to 4.5 wt%). The materials were prepared using a co-rotating twin-screw microextruder (DSM Xplore 5&15 Micro Compounder, Sittard, The Netherlands). Films were produced in the form of coils by co-extrusion in a twin-screw extruder equipped with a 6–7 cm width flat head. The detailed processing conditions are listed in Table S1.

### 2.3. Sample Characterization

*Infrared Spectroscopy*. The presence of functional groups in the samples was studied using a Thermo Scientific Nicolet iS20 FTIR Spectrometer (Waltham, MA, USA), performing a total number of 64 scans per sample in the frequency range of 4000–600 cm^−1^ at room temperature and humidity conditions. Samples were dried prior to analysis for a minimum of 48 h.

*Scanning Electron Microscopy Test.* The microstructural features of the virgin material and of the nanocomposites were investigated by a Field Emission Scanning Electron Microscope (FE-SEM), Zeiss model Supra 25 (Carl Zeiss AG, Feldbach, Switzerland), using secondary electrons and an accelerating voltage of 1 kV. Before the observations, the samples were sputter-coated with gold for 40 s to make the specimens electrically conductive. SEM images were analyzed to determine the change in morphology before and after the addition of different weight percentages of the masterbatch containing multi-walled carbon nanotubes. The analysis was carried out on cryo-fractured surfaces of samples obtained from cuts parallel and perpendicular to the drawing direction.

*Thermogravimetric Analysis*. To evaluate the degradation and decomposition temperature characteristics of the materials, thermogravimetric analysis was performed with a Perkin Elmer TGA 4000 (Waltham, MA, USA) under nitrogen flow at 40 mL/min. The temperature program included a single heating scan step ranging from 40 °C to 800 °C at 10 °C/min. The samples subjected to TGA were previously dried and weighed directly in the appropriate pan using a KERN ABT 220-5DNM analytical balance (Balingen-Frommern, Germany). The maximum degradation temperature (T_max_) was calculated as the minimum of the thermogram derivative, while the onset temperature (T_onset_) was calculated in tandem with the weight loss onset.

*Differential Scanning Calorimetry*. The thermal properties of the investigated materials were evaluated using Differential Scanning Calorimetry (DSC) with a Pyris DSC6 instrument (Waltham, MA, USA) under nitrogen flow at 20 mL/min. The temperature program was set as follows: (i) isotherm for 1 min at 0 °C, (ii) 1st heating scan ranging from 0 °C to 300 °C at 10 °C/min, (iii) isotherm for 1 min at 300 °C, (iv) cooling scan ranging from 300 °C to 0 °C at 100 °C/min, (v) isotherm for 10 min at 0 °C, and (vi) 2nd heating scan ranging from 0 °C to 300 °C at 10 °C/min. The samples subjected to DSC were prepared by weighing 5–15 mg range of previously dried material directly into the appropriate pan using a KERN ABT 220-5DNM analytical balance (Balingen-Frommern, Germany). T_g_ was calculated as the midpoint of the heat capacity increment related to the glass-to-rubber transition, while Tm was considered as the peak value of the endothermal phenomenon in the DSC curve. Δc_p_ was calculated from the vertical distance between the two extrapolated baselines at T_g_, while ΔH_m_ was calculated considering the area subtended by the melting peak.

*Tensile Mechanical Tests*. The mechanical performances of the samples were evaluated using Instron 5966 apparatus (Norwood, MA, USA) with manual grips for thin films. The tensile speed (10 mm/min) was applied on 5 × 50 mm specimens with a gauge length of 20 mm at temperature and humidity conditions in the ranges of 23–25 °C and 60–70%, respectively. A minimum of 5 specimens were tested for each sample, coming from coiled materials both from parallel cuts and cuts perpendicular to the stretching/gathering direction of the coil. From the stress–strain graphs, it was possible to calculate the average values of Young’s modulus (E), tensile strength (σ_max_), and elongation at break (Ɛ_B_).

*Surface Wettability Tests*. The surface wettability of the materials was studied with a Drop Shape Analyzer DSA-30 (Hamburg, Germany) using distilled water under room temperature and humidity conditions. A total of 4 µL drops was dispensed at a rate of 100 µL/min directly onto each sample (10 × 60 mm films). The images used for the calculation of the contact angle in water were taken 5 s after drop deposition. The contact angle (θ) was calculated as the angle formed by the interface lines between solid–liquid and liquid–vapor.

*Electrical Properties Test.* The volumetric resistivity of polymeric materials was evaluated using a source meter unit coupled to a Test Fixture. The samples were produced by cutting circular specimens with a diameter of 65 mm. A voltage of 200 V was applied, and after 2 s, the value of the current used to calculate volumetric resistivity was recorded. The volumetric resistivity (ρ) was calculated using Equation (1):

(1) $$\rho =\frac{22.9\;[V]}{tc\left[\mathrm{cm}\right]•I[A]}=[\Omega •\mathrm{cm}]$$

where ***tc*** is the sample’s thickness in centimeters, ***I*** is the measured current in amperes, and **22.9** is a conversion factor in volts used to normalize measurements based on the voltage applied by the instrument.

*Electro-Mechanical Properties Test.* Specimens were cut into 10 mm large strips and inserted in a tensile machine (LLOYD Instruments LR30 K mechanical testing machine, Bognor Regis, UK) coupled with an electrometer. The grips were modified to make them electrically non-conductive. To further avoid the effect of the electrodes’ weight on the mechanical properties of the specimens, the positive and negative poles were attached on the two free ends of the specimens that stick out from the grips. A voltage of 10 V was applied, and the values of the current were registered with a Keithley electrometer, model 6517B (Solon, OH, USA). Prior to electrical conductivity measurements, each specimen was subjected to the applied voltage until a steady-state current was reached. In fact, immediately after voltage application, the measured current exhibited an initially higher value, followed by a gradual decrease with time before stabilizing. This transient behavior is commonly attributed to polarization phenomena and charge accumulation within the composite, particularly at the interfaces between the polymer matrix and the conductive fillers, as well as to charge-trapping processes [29,30]. Once these transient effects have decayed, the current reaches a nearly constant value, representative of the intrinsic conductive behavior of the material. Therefore, all conductivity values reported in this work were determined only after steady-state conditions were achieved. The electrical current was measured simultaneously with the deformation of the specimen, both in quasi-static tensile tests (deformation control) and in the cyclic mode (deformation control).

For the tensile tests, a load cell of 50 N was used. Tests were carried out on the materials containing the highest amount of CNTs (4.5 wt%) in order to evaluate the material over the percolation threshold. Static tensile tests were carried out using the following parameters: pre-load: 0.1 N, rate: 5 mm/min, area of the coupon: 10 mm × 0.13 mm, and gauge length: 30 mm. Cyclic tensile tests were carried out using the following parameters: pre-load: 0.1 N, rate: 5 mm/min, area of the coupon: 10 mm × 0.13 mm, gauge length: 30 mm, number of cycles: 10, and cyclic deformation: ranging from 1 mm to 3 mm (mean value of deformation: 2 mm).

## 3. Results

**Figure 2:**
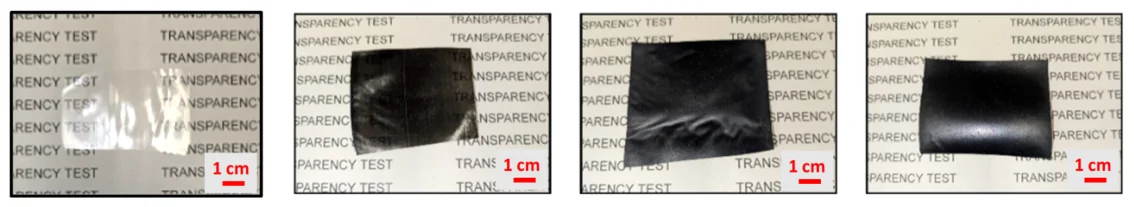
Pictures of coil samples. From left to right: Sipolprene; Sipolprene + 10% Plasticyl; Sipolprene + 20% Plasticyl; Sipolprene + 30% Plasticyl.

The obtained nanocomposites were designated as Sipolprene + X% Plasticyl, where X corresponds to the weight percentage of the masterbatch used in the specific formulation, ranging from 10 wt% to 30 wt% relative to the virgin polymeric matrix. The neat masterbatch was not extruded because its high brittleness did not allow its extrusion with the same parameters employed for the other samples [31]. Coils were obtained with a width equal to that of the die (65 mm) and a thickness of around 90 ± 10 µm, from which specimens were obtained. All coiled materials exhibit excellent processability and machinability, making them suitable for potential industrial applications. Moreover, they appear as homogeneous films with a dark color imparted by the presence of carbon nanotubes, except for neat SIPOLPRENE, which appears as a thin, colorless, and transparent film (Figure 2).

### 3.1. Molecular Characterization—FT-IR Spectroscopy

To analyze and investigate the functional groups of the materials under study, as well as to assess the possible modification of the functional groups of the polymer matrix due to the addition of the filler, FT-IR measurements were performed. The obtained spectra (Figure S1) confirmed the presence of functional groups characteristic of the Sipolprene matrix. Indeed, the characteristic absorption bands of aliphatic groups (–CH_2_–, –CH_3_) can be observed in the 2850–2950 cm^−1^ range, corresponding to the stretching vibrations of C–H bonds, along with the absorption band of the carbonyl group in the range of around 1720–1750 cm^−1^. At around 1460 cm^−1^, a signal associated with CH_2_ bending and asymmetric CH_3_ bending is present, while at approximately 1375 cm^−1^, the symmetric deformation of the CH_3_ group is detected. Finally, the absorption band related to the stretching of the ester C–O bond spans the 1100–1300 cm^−1^ range, with a main peak near 1230 cm^−1^.

As for the nanocomposites, variations in the intensity and position of the main bands can be observed, suggesting the occurrence of physical interactions between the filler and the matrix, as will be further confirmed by the thermal and mechanical analyses discussed below. More specifically, the relative FT-IR spectra show a significant band in the 1570–1600 cm^−1^ range, which can be attributed to the stretching of C=C double bonds in aromatic structures typical of carbon nanotubes [32]. An additional band around the 1700–1725 cm^−1^ range is associated with the stretching of carbonyl groups, while the band at approximately 3400 cm^−1^ can be attributed to O–H stretching vibrations, characteristic of hydroxyl groups, the intensity of which increases proportionally with the filler content. These observations are consistent with literature data reported for similar systems based on MWCNTs and polyesters.

### 3.2. Morphological Characterization

**Figure 3:**
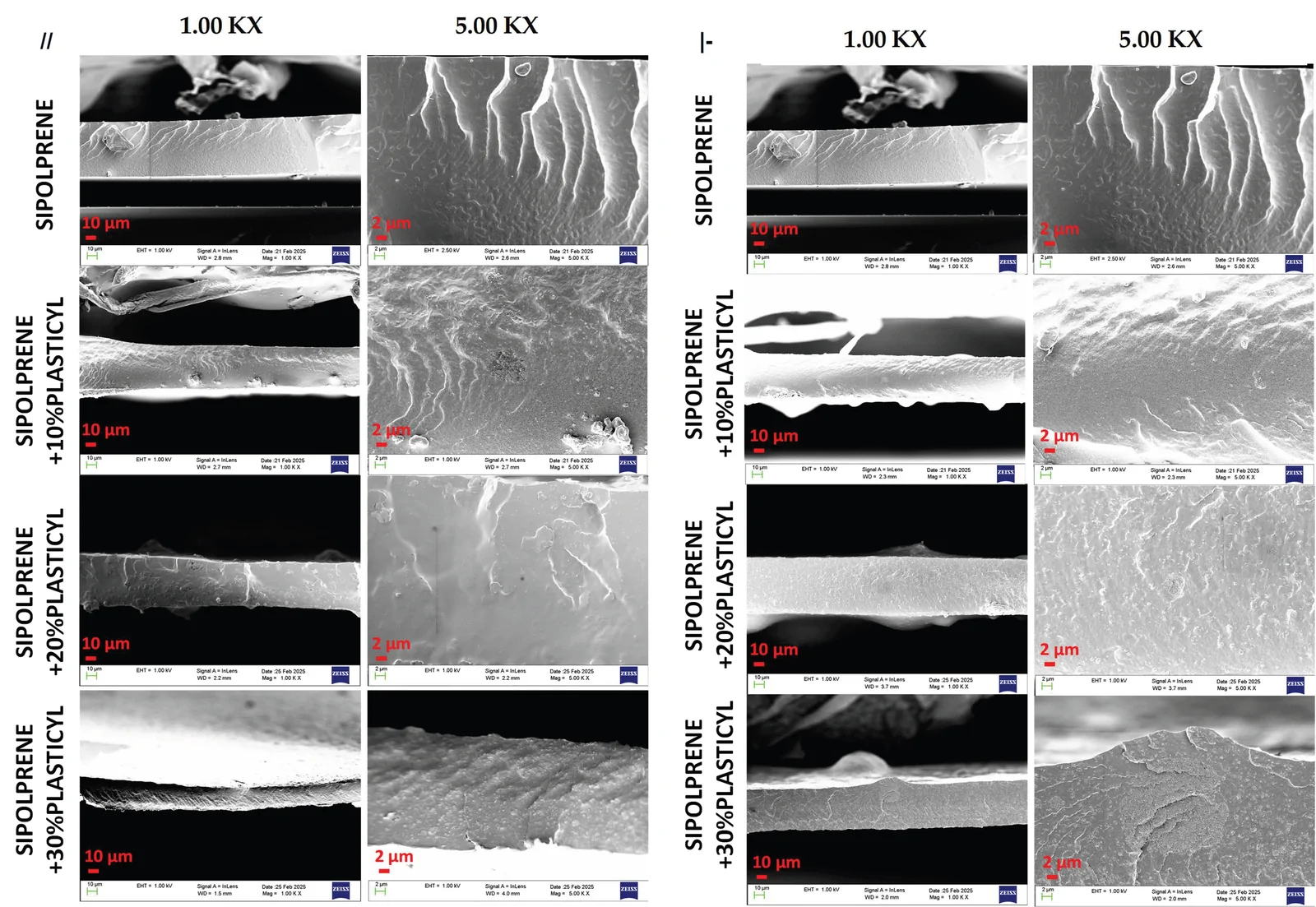
SEM images of cryo-fractured surfaces of samples obtained from cuts parallel (**left**) and perpendicular (**right**) to the drawing direction.

The samples were morphologically characterized by scanning electron microscopy, to evaluate the efficient dispersion of the filler inside the polymeric matrix. The fracture surfaces were examined at different magnifications, starting from specimens obtained from both parallel and perpendicular cuts (Figure 3) with respect to the collection direction of the coils during the extrusion process.

As can be observed from the SEM images at lower magnifications (1 KX), all sections appear smooth and free of significant discontinuities or defects, indicating the good dispersion of the filler within the Sipolprene matrix. This characteristic is a necessary condition to ensure satisfactory functional properties. Moreover, if higher magnifications (5 KX) are considered, the surfaces are smooth and defect-free, confirming the high compatibility between the two components of the nanocomposites.

### 3.3. Thermal Characterization

**Figure 4:**
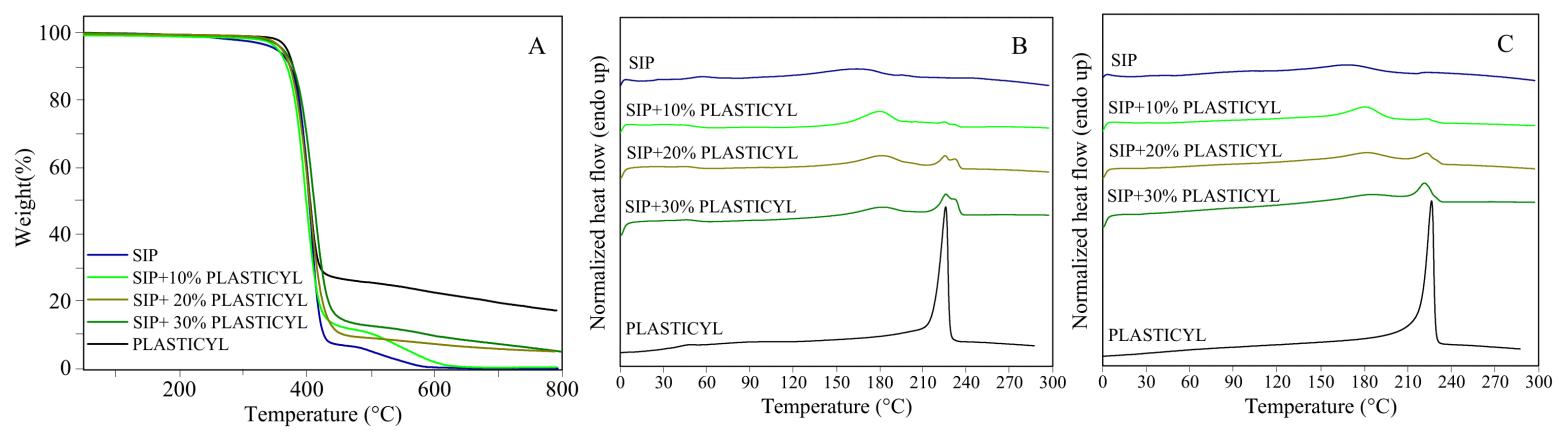
TGA (**A**), I scan DSC (**B**), and II scan DSC (**C**) curves of Sipolprene, Plasticyl, and their nanocomposites.

To evaluate the maximum temperature that should not be exceeded during processing, the thermal stability of Sipolprene, Plasticyl, and the relative nanocomposites was evaluated through TGA analysis. The obtained thermograms are shown in Figure 4A, while the values of T_onset_ and T_max_ are listed in Table 2.

Both Sipolprene and the masterbatch exhibit high and comparable thermal stability, with a T_onset_ range of 380–381 °C and T_max_ value of 403 °C. Moreover, the degradation profiles show a two-step process for the polymer matrix and a single-step process for the masterbatch. In the latter case, a residual char of approximately 20% can also be observed, consistent with the content of carbon nanotubes. The incorporation of MWCNTs into the polymeric matrix results in comparable or just slightly lower thermal stability with respect to the two reference materials. More specifically, the sample containing 10 wt% Plasticyl exhibits the lowest thermal stability, with a T_onset_ value of 371 °C and T_max_ value of 392 °C (Table 1). However, as the nanotube content increased, a slight improvement in thermal stability was observed, reaching values comparable to those of the reference polymer matrix and the masterbatch for the composite containing the highest filler amount. This suggests that higher amounts of nanotubes may exert a stabilizing effect. Regarding the degradation profiles and residual char observed in the composite samples, they are all consistent with the nanotube content. In particular, the sample containing 10 wt% of Plasticyl shows a two-step degradation profile similar to that of the neat polymer, with complete weight loss at 800 °C. In contrast, the compositions with a higher Plasticyl content exhibit a single-step degradation profile with a residual char of approximately 10%, in agreement with the behavior observed for the neat filler. The obtained results are consistent with those reported in the literature for polymer nanocomposites based on MWCNTs [14], confirming that a homogeneous dispersion of nanotubes within the matrix can result in high thermal stability.

All materials were also subjected to calorimetric analysis to evaluate their main thermal transitions, which are listed in Table 2.

By examining the first heating scan profiles (Figure 4B), it can be observed that Sipolprene is a semicrystalline material, characterized by a relatively broad and slightly intense endothermic melting peak at 165 °C, associated with the melting of PBT crystals. The position of this peak is shifted to lower temperatures compared to that of neat PBT, indicating crystals with a lower degree of perfection. Moreover, its relatively low intensity (ΔH_m_ = 14 J/g) suggests that the crystallinity of the polymer is limited by the presence of the polyether block. Conversely, the endothermic step in the baseline associated with the glass transition of the polyether segment occurs at a very low temperature (−65 °C), as confirmed by the technical datasheet, and is therefore not detectable under the adopted experimental conditions. As for the masterbatch, it behaves as a glassy and semicrystalline material, with a T_g_ value of 42 °C and a sharp endothermic peak at 228 °C, corresponding to the melting of PBT, in which the nanotubes are dispersed.

If the nanocomposites are considered, an intermediate behavior between the two reference materials can be observed, in line with their composition (Table 2). More specifically, two endothermic peaks are detected, corresponding to the melting of the polymeric matrix and the masterbatch, respectively. In addition, the former is shifted to a slightly higher temperature compared to neat SIPOLPRENE, likely due to a refinement of the crystalline phase induced by the presence of nanotubes, which act as nucleating agents and promote the formation of more perfect crystals. Regarding the intensity of this phenomenon, a slight increase in ΔHm was observed for the sample containing 10 wt% Plasticyl, while an opposite trend was observed for higher filler contents: it is reasonable that a low amount of MWCNTs (≈1.5 wt%) may promote PBT crystallization, while higher contents (3–4.5 wt%) may hinder it. As for the melting peak associated with the masterbatch, its intensity is proportional to the composition of each nanocomposite too, although its position remains essentially unchanged. Interestingly, this peak appears as two almost overlapped endothermic signals, likely due to the melting–crystallization–melting processes of the PBT crystalline phase. With regard to glass-to-rubber transitions, only those associated with PBT inside the masterbatch are detectable, although with low intensity (Table 2).

The second DSC heating scan (Figure 4C) was performed after rapid cooling from the melt, to inhibit crystallization as much as possible and allow a better evaluation of the glass transition. As can be observed, all materials exhibit profiles similar to those obtained in the first scan, meaning that the adopted experimental conditions were not sufficient to fully suppress crystallization. Moreover, also in this case, for the nanocomposites, only the glass transition associated with the PBT phase is observable, while the transition related to SIPOLPRENE^®^ occurs at temperatures too low to be detected under the applied experimental conditions, as already observed in the first scan.

As summarized in Table 2, the thermal parameters measured led to the conclusion that both the Sipolprene matrix and the nanotube source constituted by Plasticyl can be processed in the melt in a temperature range that exceeds the melting temperature of the lowest melting agent (Sipolprene) but remains well below the degradation temperature of the component most susceptible to degradation (Plasticyl).

### 3.4. Mechanical Characterization

**Figure 5:**
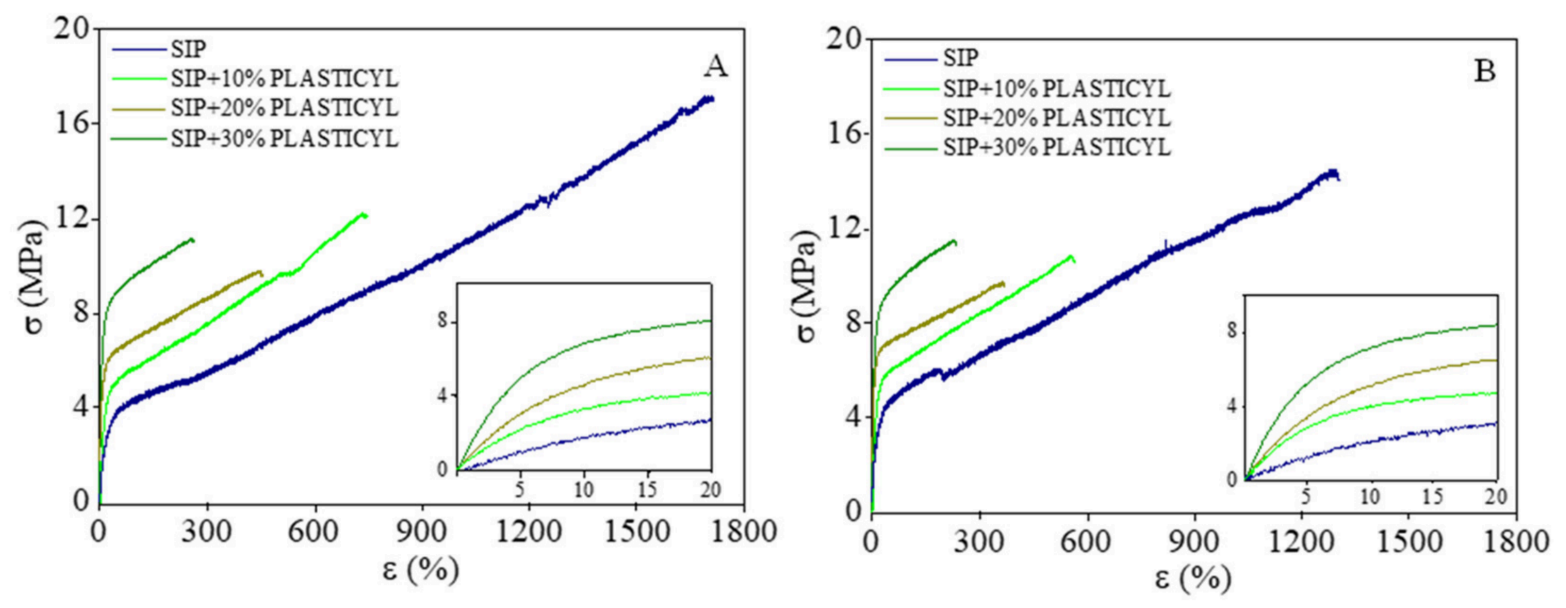
Stress–strain curves of samples obtained from cuts parallel (**A**) and perpendicular (**B**) to the drawing direction of the coil.

The study of the mechanical response was performed through tensile tests. The relative stress–strain curves, obtained from cuts parallel and perpendicular to the drawing direction of the coil, are shown in Figure 5, while the corresponding mechanical characterization data are listed in Table 3.

Mechanical tests confirmed that Sipolprene is a highly flexible material, characterized by an elastic modulus in the range of about 25–28 MPa and a very high elongation at break, over 1000%, for both the tested directions. Conversely, Plasticyl could not be tested due to its extreme rigidity and brittleness. As for the formulations, a notable increase in Young’s modulus and a parallel decrease in elongation at break were observed in all cases, indicating an increase in rigidity resulting from the addition of carbon nanotubes. As for the maximum stress, it slightly decreased for all the samples compared to Sipolprene, although it remained relatively high for all compositions. The increase in Young’s modulus with increasing MWCNT content can be attributed to the intrinsically high stiffness of carbon nanotubes, whose Young’s modulus is typically around a range of 1–4 TPa [4]. When uniformly dispersed within the polymer matrix, MWCNTs act as highly efficient reinforcing elements that restrict the mobility of polymer chains and improve stress transfer from the matrix to the nanotubes through the filler–matrix interface. As the filler concentration increases, the average distance between nanotubes decreases, promoting the formation of an interconnected reinforcing network that further limits the deformation of the polymer under load [33].

By comparing the samples analyzed along the direction parallel to drawing with those tested in the perpendicular direction (Figure 5, Table 3), it can be observed that, by keeping the composition fixed, the former are slightly less rigid and more flexible, with lower E values and higher ε_B_ values. This trend is consistent across all nanocomposites, except for the sample containing the highest number of carbon nanotubes, for which the mechanical data are practically the same. Of note, all the nanocomposites remain easy to handle and quite deformable, two important characteristics in view of potential technological applications. Overall, the collected data confirm the good compatibility between the polymer matrix and the filler, in agreement with SEM analysis (Figure 3).

### 3.5. Surface Wettability—WCA Measurements

The evaluation of the hydrophobic character of the polymer films was performed through WCA measurements. Images of water drops on polymer surfaces are shown in Figure S2, while the corresponding WCA values are listed in Table 4. Both SIPOLPRENE and Plasticyl are materials with a hydrophobic level (WCA values of 93° and 99°, respectively). The nanocomposites showed behavior contrasting the two reference materials, resulting quite hydrophilic: in particular, the lower the amount of nanotubes inside the formulation, the greater the hydrophilicity. This trend suggests a modification in the surface tension of the material, due to the complex interaction between the nanotubes and the matrix in which they were dispersed. It is also important to note that the presence of nanotubes within the nanocomposites leads to a non-negligible increase in surface roughness (Figure S2), which may have significantly influenced the measurement [18].

### 3.6. Electrical Properties

All materials investigated in this work were subjected to volume electrical resistivity measurements in order to evaluate the electrical conductivity of the produced nanocomposites in comparison with the virgin matrix. The analysis was carried out at room temperature on circular samples with a diameter of 65 mm. The obtained values are reported in Table 4. As expected, Sipolprene was characterized by high resistivity (Table 4), typical of insulators. Similar behavior was observed for the formulations with less filler, although an increase in the content of carbon nanotubes was responsible for a progressive reduction in the volumetric resistivity values, i.e., improved electrical conductivity [34]. Of note, the sample containing 30% Plasticyl showed the typical behavior of a conductive material, making it suitable for potential use in electronic applications. The electrical resistivity of pure Plasticyl was not measured because, although these pellets can be processed into thin films, the resulting films are highly brittle and prone to fracture during handling, making them unsuitable for reliable electrical resistivity measurements.

**Figure 6:**
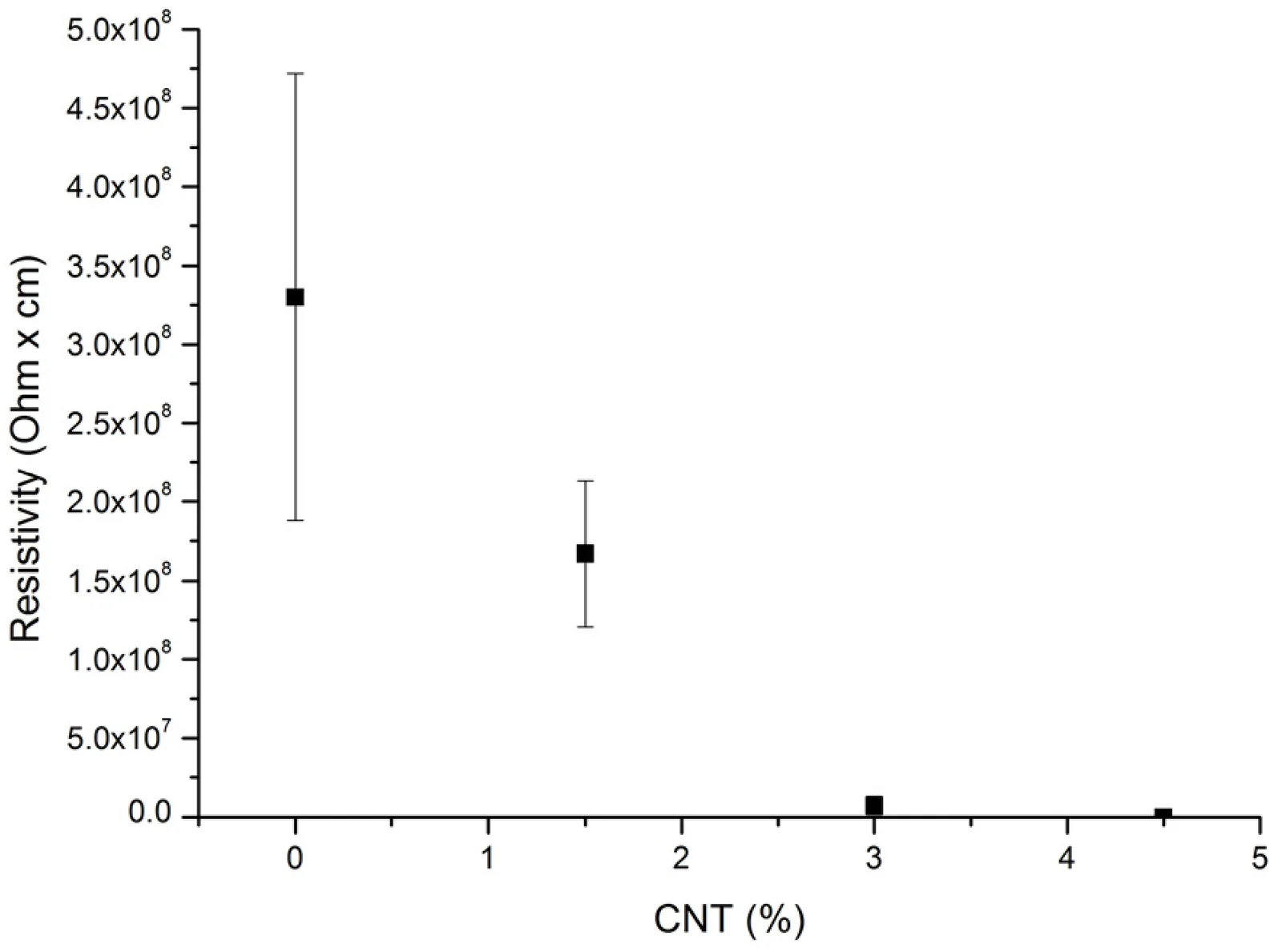
Volume resistivity as a function of CNT content.

In Figure 6, the plot of volume resistivity as a function of CNT content (wt%) allows the percolation threshold to be readily identified. In fact, the electrical resistivity decreases only moderately, from 10^8^ to 10^6^ Ω·cm, when the CNT content increases from 1.5 wt% to 3 wt%. In contrast, a dramatic drop to approximately 10^−3^ Ω·cm is observed at 4.5 wt%, indicating that the percolation threshold is located between 3 and 4.5 wt%.

The pronounced decrease in volume resistivity with increasing MWCNT content is associated with the formation of electrically conductive pathways inside the polymer matrix [35]: at low filler concentrations, carbon nanotubes remain isolated and the polymer behaves as an electrical insulator. As the MWCNT content increases, the average distance between adjacent nanotubes decreases until the electrical percolation threshold is reached. Beyond this threshold, an interconnected conductive network is established, allowing electron transport through direct contacts and tunneling mechanisms between neighboring nanotubes. As a result, the electrical resistivity decreases by several orders of magnitude. The composite containing 30 wt% of masterbatch (4.5 wt% of CNTs in the final material) is well above the percolation threshold and therefore exhibits stable electrical conductivity due to the presence of a dense conductive network. Such a continuous network is advantageous for sensing applications because mechanical deformation modifies the contact resistance between adjacent nanotubes and the tunneling distances within the network, producing measurable changes in electrical resistance (piezoresistive effect). At the same time, a sufficiently interconnected conductive network provides stable baseline conductivity, which is essential for obtaining reproducible sensor responses.

Although the percolation threshold observed in the present study is higher than that reported in several previous works [19,36,37], it is well established that the electrical percolation threshold of CNT/polymer composites is not an intrinsic property of the nanotubes themselves but it is ruled by a combination of factors, including the dispersion state of the CNTs, their aspect ratio, the type (single wall or multi-wall), the processing route, the polymer matrix characteristics, nanotube alignment, interparticle tunneling distance, CNT–polymer interfacial interactions, and defects on the CNT surface. In addition, in the present work, the MWCNTs were not dispersed directly into the Sipolprene matrix but were introduced through a Plasticyl masterbatch. Therefore, the electrical response may also depend on the compatibility and dispersion of the Plasticyl phase within the Sipolprene matrix, which may affect the redistribution of the nanotubes and, consequently, the formation of a continuous conductive network [38].

**Figure 7:**
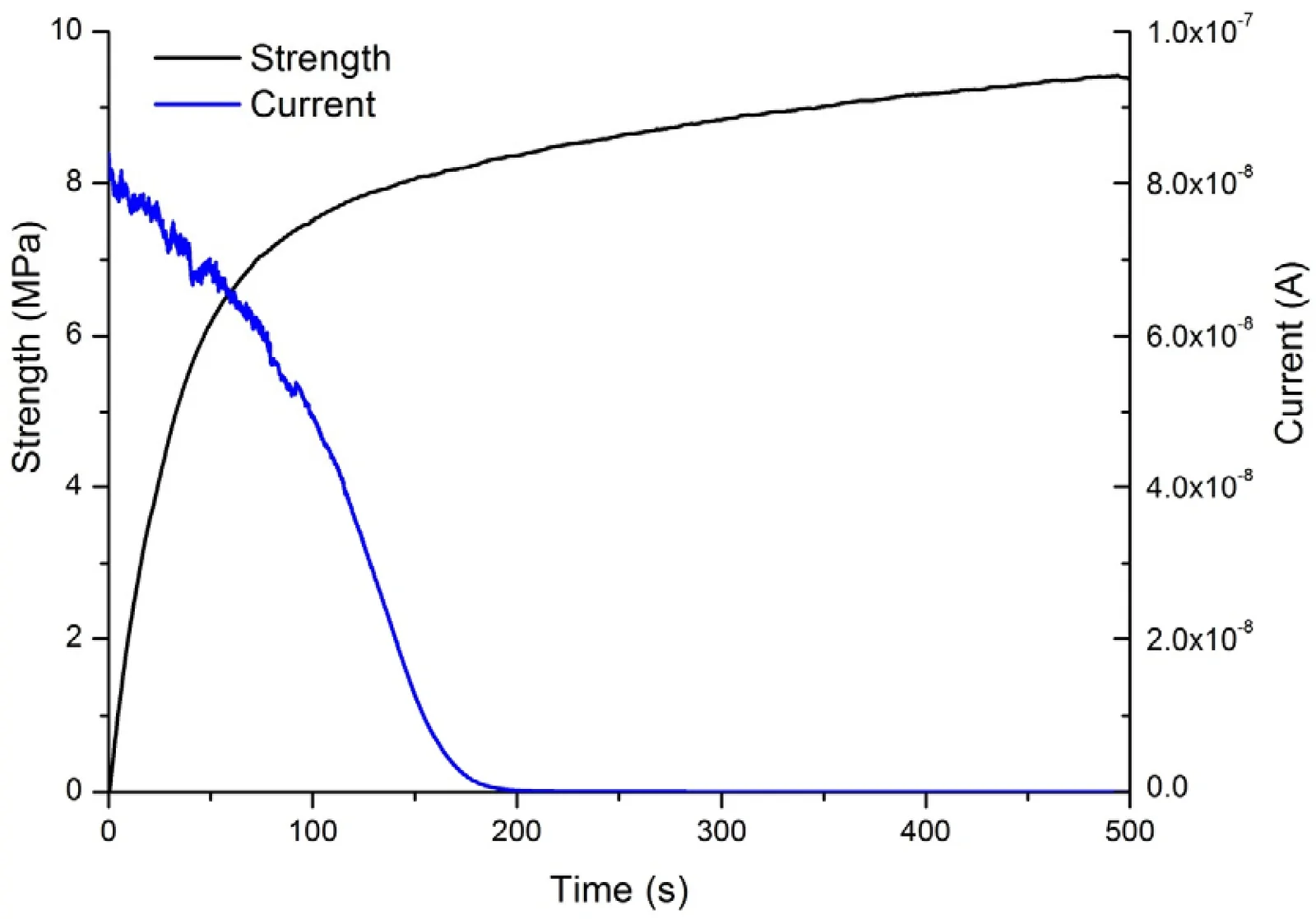
Evolution of stress and current as a function of time in a tensile quasi-static test.

In Figure 7, the static electro-mechanical test carried out on the specimen containing the highest amount of CNTs is shown: the evolution of mechanical strength and current as a function of time is reported.

It is possible to detect that the current tends to decrease with the stretching of the specimen; in fact, during elongation, the distance among the conductive domains increases with a consequent reduction in the current [39]. Generally, the electrical conduction is interrupted when cracks or fractures occur in the specimen [40] breaking the conduction path. On the other hand, as reported in Figure 6, in the case of Sipolprene, the current continuously decreases with a rapid drop to 0 after the yield point and long before the fracture occurs. This suggests that conductive path breakage is not due to the formation of cracks or voids within the material, but it is probably caused exclusively by the increasing separation of the conductive domains, which reaches its maximum value after the yield point, i.e., when plastic deformation begins. This is an interesting result because it highlights that the material exhibits electrical piezoresistivity only before its plastic deformation.

**Figure 8:**
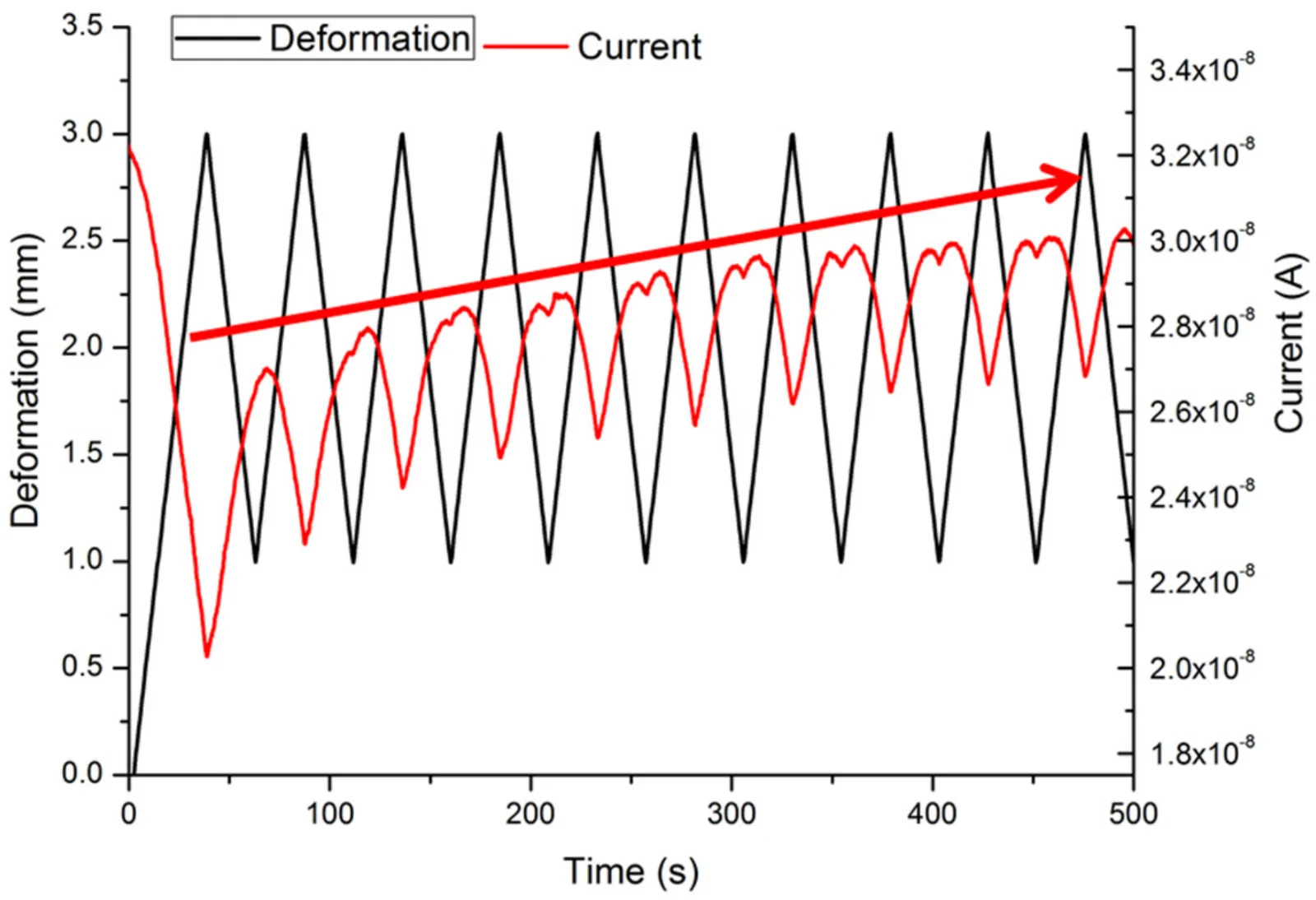
Evolution of deformation and current as a function of time in a tensile cyclic test.

In Figure 8, the electro-mechanical test in the dynamic mode is reported: the coupon is subjected to cyclic deformation from 1 mm to 3 mm. In this case, the mean value of the deformation is 2 mm and the amplitude is 1 mm.

Figure 8 highlights that, in cyclic load tests, the conductive film can successfully register in real time the deformation occurring in the specimen. It is possible to highlight that the minimum current value corresponds to the maximum deformation value and, vice versa, the same reason is exhibited for the static test: during cyclic deformation, a piezoresistive response was observed, with the electrical current decreasing during the loading stage and increasing during unloading. This behavior is commonly attributed to the increase in the average distance between neighboring conductive particles. It is interesting to note that the current value tends to increase with time (red arrow) even if the specimen is affected by increasing the voltage to reach a constant current value before the test. This effect suggests that, in addition to the reversible changes associated with the applied strain, the conductive network undergoes a gradual microstructural rearrangement during cyclic loading, probably due to a local reorganization of the conductive fillers within the viscoelastic polymer matrix, potentially leading to the formation of more efficient electrical contacts between adjacent conductive particles. As a result, the overall electrical conductivity of the composite may increase progressively during the initial stages of cyclic loading. This interpretation is consistent with the previous observation made for the quasi-static test in which, under monotonic tensile loading, the electrical current rapidly decreased and approached zero once the material exceeded its yield point indicating an irreversible disruption of the conductive network. In contrast, under the cyclic deformation levels, the conductive pathways appear to remain largely intact (the material was operating in its elastic region), while it undergoes a progressive reconfiguration toward a more stable and electrically conductive state [41,42].

## 4. Conclusions

Nanocomposites based on Sipolprene 25170-W reinforced with 10, 20, and 30 wt% of Plasticyl, corresponding to MWCNT content values of 1.5 wt%, 3 wt%, and 4.5 wt%, exhibited properties strongly dependent on nanotube content. The main experimental findings can be summarized as follows:

- Increasing the MWCNT content progressively increased the Young’s modulus, indicating enhanced stiffness, while reducing the elongation at break. Despite this, all formulations maintained good processability and handleability. This suggests both good compatibility between the matrix and the filler, as confirmed by SEM analysis, and that the incorporation of MWCNTs within the polymeric matrix can improve structural resistance while preserving flexibility.
- SEM observations revealed a homogeneous dispersion of MWCNTs within the polymer matrix, suggesting an effective filler–matrix interaction and efficient stress transfer.
- Water contact angle analysis revealed a more pronounced hydrophilic behavior of the nanocomposites compared to the neat Sipolprene, which may be advantageous in applications where an interaction with humid environments or polar substances is critical.
- Increasing MWCNT loading produced a progressive reduction in volume resistivity. At the highest filler content (30 wt% of Plasticyl, 4.5 wt% of MWCNTs), a continuous conductive network was established, resulting in electrically conductive behavior.
- Static and dynamic electro-mechanical tests demonstrated a stable correlation between electrical resistance (or current) and applied deformation, confirming the suitability of the nanocomposites for in situ strain-sensing applications demonstrating a stable and measurable piezoresistive response.

Finally, the use of a commercial masterbatch containing MWCNTs pre-dispersed in a polymer matrix represents a practical processing strategy, facilitating nanotube dispersion while reducing handling issues associated with pristine carbon nanotubes. This approach improves process safety and makes the manufacturing route more attractive for industrial scale-up. Overall, the combination of improved mechanical performance, tunable electrical conductivity, and reliable piezoresistive behavior demonstrates the potential of these nanocomposites for advanced applications, including flexible sensors, electronic devices, and electromagnetic interference (EMI) shielding systems.

## Figures and Tables

**Table 1:** Main technical data of Sipolprene25170-W, Plasticyl PBT-1501, and NC7000 from the relative technical datasheets.

**Sipolprene**	Flexural modulus (MPa)	Strain at break (%)	MFI (g/10 min)	T_m_ (°C)		
	30	>300	12	173		
**Plasticyl**	MWCNTs loading (%wt)	Real density (g/L)	MFI (g/10 min)	T_m_ (°C)		
	15 ± 1	1300	0.88	227		
**NC7000**	Average diameter (nm)	Aspect ratio	Specific Surface Area (m^2^/g)	Volume resistivity (Ω cm)	Thermal conductivity (W/mK)	Tensile strength (GPa)
	9.5	100–1000:1	250–300	10^−4^	>3000	10–60

**Table 2:** Thermal characterization (TGA and DSC) data of the materials under study.

Sample	TGA		I Scan DSC				II Scan DSC			
	T_onset_ [°C]	T_max_ [°C]	T_g_ [°C]	Δc_p_ [J/g °C]	T_m_ [°C]	ΔH_m_ [J/g]	T_g_ [°C]	Δc_p_ [J/g °C]	T_m_ [°C]	ΔH_m_ [J/g]
Sipolprene	381	403	n.d.	n.d.	165	14	n.d.	n.d.	165	12
Sip + 10% Plasticyl (1.5 wt% of CNTs)	371	392	40	0.054	180 224	18 2	40	0.024	180 224	11 1
Sip + 20% Plasticyl (3 wt% of CNTs)	375	401	40	0.037	181 226	13 5	41	0.017	181 223	10 4
Sip + 30% Plasticyl (4.5 wt% of CNTs)	379	404	41	0.073	182 227	8 9	41	0.045	182 222	6 6
Plasticyl	380	403	42	0.034	228	42	42	0.344	228	37

**Table 3:** Data from static tensile mechanical tests for parallel and perpendicular cuts.

Sample	E [MPa] ║ Cut	E [MPa] ┴ Cut	σ_max_ [MPa] ║ Cut	σ_max_ [MPa] ┴ Cut	ε_B_ [%] ║ Cut	ε_B_ [%] ┴ Cut
Sipolprene	25 ± 1	28 ± 2	15 ± 3	15 ± 4	1751 ± 173	1442 ± 172
Sip + 10% Plasticyl (1.5 wt% of CNTs)	45 ± 2	70 ± 9	12 ± 1	10 ± 1	745 ± 66	526 ± 72
Sip + 20% Plasticyl (3 wt% of CNTs)	58 ± 7	84 ± 6	9 ± 2	9 ± 1	423 ± 34	298 ± 61
Sip + 30% Plasticyl (4.5 wt% of CNTs)	122 ± 6	122 ± 11	11 ± 1	11 ± 1	263 ± 16	264 ± 15

**Table 4:** Values of water contact angle (WCA) and volumetric resistivity measured for the polymer matrix, the masterbatch, and the corresponding nanocomposites.

Sample	WCA [°]	Volumetric Resistivity [Ohm × cm]
Sipolprene	93 ± 4	3.30 × 10^8^ ± 1.42 × 10^8^
Sip + 10% Plasticyl (1.5 wt% of CNTs)	72 ± 2	1.67 × 10^8^ ± 4.61 × 10^7^
Sip + 20% Plasticyl (3 wt% of CNTs)	78 ± 2	7.20 × 10^6^ ± 5.62 × 10^6^
Sip + 30% Plasticyl (4.5 wt% of CNTs)	81 ± 2	2.31 × 10^−3^ ± 1.32 × 10^−5^
Plasticyl	99 ± 4	-

## Data Availability

The original contributions presented in this study are included in the article/Supplementary Material. Further inquiries can be directed to the corresponding author.

## Supplementary Materials

The following supporting information can be downloaded at: https://www.mdpi.com/article/10.3390/ma19163397/s1; Figure S1: FT-IR spectra of the neat polymer matrix, the filler, and the nanocomposites; Figure S2: Pictures of water droplets on the surfaces of the neat polymer matrix, the filler, and the nanocomposites; Table S1: Process parameters for melt compounding and cast film production.
