# Supplementary material for: Investigation of the Physical Properties of Poly(ester–ether)s and Multi-Walled Carbon Nanotube Nanocomposites
Source: Materials (Basel). 2026 Aug 10;19(16):3397. doi: 10.3390/ma19163397 (PMC13514636; doi:10.3390/ma19163397)
Supplement: Supplementary file 1 [file materials-19-03397-s001.zip › materials-4478593-supplementary.pdf]

Supporting Information

Table S1. Process parameters for melt compounding and cast film production.

**Formulation**	**Matrix**	grade	Sipolprene 25170	Sipolprene 25170	Sipolprene 25170	Sipolprene 25170
	**Copo by matrix**	%wt.	100	90	80	70
	**Masterbatch**		Plasticyl1501	Plasticyl1501	Plasticyl1501	Plasticyl1501
	**Added MB**	%wt.	0	10	20	30
	**PBT by filler**	%wt.	0	8.5	17.0	25.5
	**CNC by filler**	%wt.	0	1.5	3.0	4.5
**Melt mixing**	**Temperature**	°C	220-230-240	220-230-240	220-230-240	220-230-240
	**Permanence time**	s	180	180	180	180
	**Screw speed**	rpm	90	90	90	90
**Cast filming**	**Tdie**	°C	215	215	215	215
	**Die width**	mm	65	65	65	65
	**Force control**	N	515	565	750	1150
	**Drag speed**	mm/min	600	600	600	600
	**Winding torque**	N·mm	35	36	38	38
	**Cooling flow**	dl/min	30	30	28	26

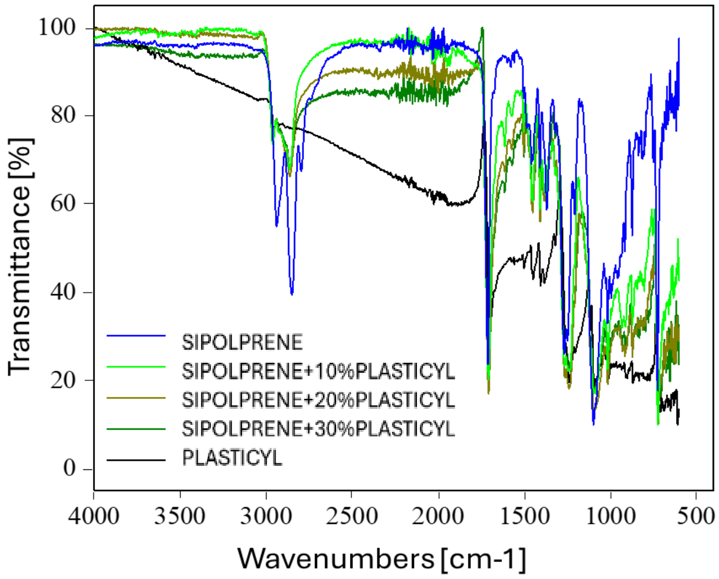

Figure S1: FT-IR spectra of the neat polymer matrix, the filler, and the nanocomposites.

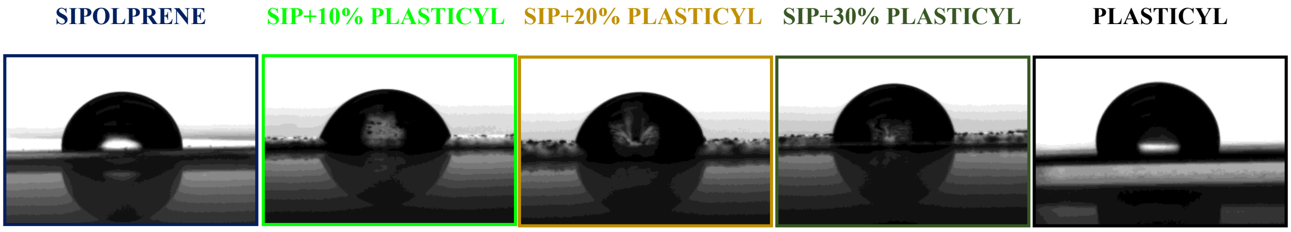

Figure S2: Pictures of water droplets on the surfaces of the neat polymer matrix, the filler, and the nanocomposites.
